# Robotic Technologies and Rehabilitation: New Tools for Stroke Patients' Therapy

**DOI:** 10.1155/2013/153872

**Published:** 2013-11-20

**Authors:** Patrizia Poli, Giovanni Morone, Giulio Rosati, Stefano Masiero

**Affiliations:** ^1^Rehabilitation Unit, Department of Neurosciences, University of Padua, Via Giustiniani 3, 35128 Padova, Italy; ^2^Clinical Laboratory of Experimental Neurorehabilitation, IRCCS Santa Lucia foundation, Via Ardeatina 306, 00179 Roma, Italy; ^3^Doctoral School, Medical Clinical and Experimental Sciences, Neurosciences, University of Padua, Italy; ^4^Department of Innovation in Mechanics and Management (DIMEG), University of Padua, Via Venezia 1, 35131 Padova, Italy

## Abstract

*Introduction*. The role of robotics in poststroke patients' rehabilitation has been investigated intensively. This paper presents the state-of-the-art and the possible future role of robotics in poststroke rehabilitation, for both upper and lower limbs. *Materials and Methods*. We performed a comprehensive search of PubMed, Cochrane, and PeDRO databases using as keywords “robot AND stroke AND rehabilitation.” *Results and Discussion*. In upper limb robotic rehabilitation, training seems to improve arm function in activities of daily living. In addition, electromechanical gait training after stroke seems to be effective. It is still unclear whether robot-assisted arm training may improve muscle strength, and which electromechanical gait-training device may be the most effective for walking training implementation. *Conclusions*. In the field of robotic technologies for stroke patients' rehabilitation we identified currently relevant growing points and areas timely for developing research. Among the growing points there is the development of new easily transportable, wearable devices that could improve rehabilitation also after discharge, in an outpatient or home-based setting. For developing research, efforts are being made to establish the ideal type of treatment, the length and amount of training protocol, and the patient's characteristics to be successfully enrolled to this treatment.

## 1. Introduction

Stroke from an ischemic or haemorrhagic intracranial vascular event is a leading cause of movement disability in the USA and in Europe [1]. The World Health Organization (WHO) estimates that in Europe stroke events will increase by 30% between 2000 and 2025 [2]. Hemiparesis/hemiplegia is the most common outcome of stroke, leading to movement deficits in the contralateral limbs to the side of the brain affected by the stroke. The main clinical characteristics observed in hemiparetic patients are: weakness of specific muscles, abnormal muscle tone, abnormal postural adjustments, lack of mobility, abnormal movement synergies, loss of joint coordination, and loss of sensitivity. The residual impaired limb function and disability in the activities of daily living (ADLs) give stroke an important social impact: the recovery is partial in stroke survivors, with 15%–30% of patients permanently disabled and 20% requiring institutional care at 3 months after onset [3].

Thus, the rehabilitation goal in poststroke subjects is to promote recovery of lost function, to allow independence and early reintegration into social and domestic life. The number of people that require rehabilitation after stroke is growing rapidly [4], with increasing costs and pressure on healthcare budgets. For example, in the USA, the direct and indirect costs of stroke in 2007 were 40.9 billion dollars, the estimated direct medical cost of stroke for 2007 was 25.2 billion dollars, and the mean lifetime cost of ischemic stroke was 140,048 dollars [3].

Poststroke patients require continuous medical care and intensive rehabilitation often requiring one-on-one manual interaction with the physical therapist. Unfortunately, present demands and budget restrictions do not allow this intensive rehabilitation. Hence, there is an urge for new technologies improving the efficacy and effectiveness of poststroke rehabilitation. The available scientific literature suggests that the most effective rehabilitative interventions are those providing early, intensive, task-specific, and multisensory stimulation. The well-known capacity of the central nervous system to adapt its structural organization after brain lesion is mainly influenced by sensory input, experience, and learning [5, 6]. Nudo et al. [7] first demonstrated that subtotal lesion, confined to a small portion of the representation of one hand in adult squirrel monkeys, yields a further loss of hand territory in the adjacent undamaged cortex that could be successfully prevented by the retraining of the skilled hand. Thus, there is increasing evidence that the motor system is plastic following stroke and can be influenced by motor training [8]. The term “*Neural Plasticity*” indicates the recovery mechanisms and functional adaptation resulting from global changes in neuronal organization. Neural adaptation leads to a more robust recruitment of motor neuron pools, transfer of function from damaged areas to preserved adjacent or correlated areas, strengthening of redundant or parallel synapses, new synapse formation, increased dendritic sprouting, enhanced myelination of remaining neurons, and modification of cortical and noncortical representations. Recently, cerebellum has been demonstrated to play a key role in modulating cortical motor output and in motor learning [9]. Hence, although neural damage cannot be replaced by cellular proliferation, partial compensation might be provided by adaptive mechanisms, including variations in neural schemes through the unmasking of hidden neural pathways and synapses which, although not normally used, might emerge when the dominant system fails. On this basis, currently available literature advocates a strong relationship between intense multisensory rehabilitation and recovery in stroke patients. Thus, well-defined training methods-implementing intense multisensory stimulation may induce neural adaptations and enhance motor and functional recovery of the paretic upper extremity. Upon these bases, the use of automatic devices was proposed to help therapists to increase the intensity of therapies, produce a multisensory stimulation, and reduce costs during their work. This new concept dates back to the early 1990s with a new family of robotic machines called “haptic interfaces”; these mechanical devices were designed to interact with the human, by guiding the upper limb into passive and active-assisted mobilization, helping some movement tasks by biofeedback systems and measuring changes in movement kinematics and forces. Thus robotic therapy might represent a successful and standard complement for poststroke multidisciplinary rehabilitation programs.

Because of the continuous and rapid evolution of the robotic technology, the aim of the present review is to provide a comprehensive insight into the main robotic devices and their possible use in stroke patients' rehabilitation. The main devices available nowadays for upper and lower limbs rehabilitation are hereby presented. For each class of robots, areas of agreement among researchers, together with controversial aspects about their application in stroke rehabilitation are reported and discussed in the present review. Finally, the current main topics of study and areas timely for developing research are presented.

*Robotic Devices*. The robotic devices currently available can be divided into two main categories: those for the upper limb and those for the lower limb. As we previously indicated [10], the upper limb robotic systems existing until today can be classified and analysed from several points of view, as showed in Table 1.

Table 2 provides a brief summary of the main electromechanical and robot-assisted upper limb training devices cited by a recent Cochrane review [11].

The lower limb devices include exoskeletons and end-effectors and the characteristics of the main automated electromechanical gait machines are illustrated in Table 3 [12].

## 2. Materials and Methods 

*Search Methods*. The authors undertook literature search in February 2013 about robot-assisted upper and lower limbs rehabilitation after stroke, using as keywords “robot AND stroke AND rehabilitation.” The databases were PubMed/Medline, Cochrane Library, and PeDRO. Only papers written in English were considered and the search was extended to the whole database. The authors found about 300 papers, published from 1991 to February 2013.

## 3. Results and Discussion

Areas of agreement and areas of controversy among researchers emerging from the analysis of the literature on this growing field are reported.

### 3.1. Areas of Agreement

Restoring hand and arm motor function is essential to preserve patient's independence in the performance of the activities of daily living (ADLs). Rehabilitation plays a fundamental role in minimizing the residual motor deficits of stroke patients, both during the acute and subacute phases and in the chronic phase. Physicians are usually prone to prescribe treatments including intense [13], highly repetitive [14], and task-oriented [15] movements. Applying this kind of exercise to subjects with poststroke paresis results in increase in strength, accuracy, and functional use [14, 16–18]. Robotic technology represents a feasible tool to administer treatment protocols with the characteristics mentioned above. The potential of robotic systems in poststroke rehabilitation is high from different points of view. Robotic systems can be administered under the supervision of a therapist, providing an intensive, task-oriented motor training of the patient's limbs, as part of an integrated set of rehabilitation tools including also nonrobotic approaches. Robots enhance traditional poststroke treatment: they specifically provide therapy for long time periods, in a consistent and precise manner, allowing a remarkable effort and time saving, both for the patient and for the therapist; they are programmed to perform in different functional modes and automated for many functions; they can also measure and record a range of behaviours corresponding to specific therapeutic applications [19–24]. Functional recovery is fundamental for the reintegration of stroke subjects into social and domestic life, which remains the main target of rehabilitation programs; thus the robot-assisted therapy should focus on this functional goals.

The robot-assisted rehabilitation of the upper limb in the acute and subacute poststroke phases may be successfully used in alternative to conventional mobilization, resulting at least as effective as conventional therapy, especially when used in addition to nonrobotic techniques [25, 26]. Masiero et al. [27] hypothesized that an optimal robotic training protocol for acute and subacute stroke patients should be divided in two stages: initial additional robotic training (first stage) followed by substitution of part of the conventional therapy with the robotic exercise (second stage). In this way, the amount of treatment would be increased in the stage of recovery where improvements are likely to be greater. Nonetheless, from an organizational point of view, the equivalence of robotic and physical therapy may be considered as a positive result. The introduction of robotic systems into clinical practice is useful at least in promoting a cost-effective use of human resources and the standardization of rehabilitation treatments. In fact, the workload of the physiotherapist can be alleviated, allowing him to focus on the target of functional rehabilitation during individual training, and to supervise several patients at the same time during robot-assisted therapy sessions. This approach would make better use of the time and the expertise of physiotherapists, while increasing the effectiveness and efficiency of the rehabilitation program [28].

However, robotic rehabilitation is not merely a matter of increasing the amount and intensity of therapy. In fact, robotic systems may be used not only to produce simple and repetitive stereotyped movement patterns, as well as for most of the existing devices, but also to generate a more complex, controlled multisensory stimulation of the patient. It seems that rehabilitation technology impact on functional outcome could be optimized offering more chances to the nervous system to experience “real” activity-related sensorimotor input during training of upper limb movement [29]. In this way, a higher level of interaction and stimulation can be produced, with respect to the one usually experienced during a hand-over-hand therapy. Extrinsic feedback can be also offered to the patient, providing knowledge of results and/or of performance during robotic training, thus facilitating the achievement of the goal movement and promoting the enrolment of the subject in the rehabilitation exercise [30]. The improvement in motor performance in stroke patient after upper limb robot-based rehabilitation is a fact and the recent demonstration [31] that a robot-aided rehabilitation program induces brain reorganization strengthens the employment of such a technology in the rehabilitation program. Pellegrino et al. [31] observed that interhemispheric connectivity between primary somatosensory areas got closer to a “physiological level,” after a robot-assisted rehabilitation program, in parallel with the acquisition of more accurate hand control. Regarding the lower limb, the main rehabilitation goal for patients after stroke is becoming independent in walking. Like for upper limb rehabilitation, for the lower machines are available supporting the gait training. Recently treadmill training, with and without body weight support, was introduced for the rehabilitation of patients after stroke. To restore gait, most clinicians prefer a task-specific repetitive approach [32], and in recent years the better outcomes for stroke patients have been reached with repeated walking programs with growing intensities [33, 34]. However the repetitive execution of complex gait cycles for these patients requires specific devices such as the treadmill, with and without partial body weight support. Nevertheless the treadmill training requires a considerable effort by the therapist to set the paretic limbs and to control weight shift. This may limit therapy intensity especially in more severely disabled patients. In order to reduce dependence on therapists, automated electromechanical gait machines were developed. Gait machines consist either of an electromechanical solution with two driven foot plates simulating the phases of gait, or of a robot-driven exoskeleton orthotic.

The role of electromechanical devices is that, in contrast with the action of one physical therapist alone, they can provide nonambulatory patients' intensive, high repetitive, practice of complex gait cycles. Compared to treadmill training with partial body weight support, these robotic devices may reduce the effort for therapists; in fact, they no longer need to set the paretic limbs or assist trunk movements [35]. However, implementation of physiotherapy with electromechanical-assisted gait training programs in rehabilitation settings may improve walking function after stroke. The use of electromechanical-assisted gait training devices was reported to be safe and well accepted by most patients.

### 3.2. Areas of Controversy

An important target of robotic rehabilitation is to increase patients' function, activity, and participation. Although most physicians experienced of robotics consider task-oriented approaches beneficial and promising for future research [36], most rehabilitation systems support analytical training methods. To provide realistic sensorimotor input and encourage task-related problem solving, Edmans et al. [37] suggest that robotic systems should use mixed reality systems, where movement sensitive objects and machine vision create a virtual reality environment that is steered by “real” object manipulation. Unfortunately the majority of the existing robotic devices for neurorehabilitation were designed and programmed to produce simple stereotyped movement patterns, often not related to functional activities. Virtual reality that is actually used in substitution of mixed reality and/or environmental contextual training may prevent the application of training results to everyday situations. These devices always include gaming aspects, drawing the attention of the patient on what is happening during the training. Promoting an active role is another way to attract the patient's attention: technology should take into account the patient's disposition and orientation [38]. At the present time, only few applications offer enough exercise variability to support the achievement of individual targets, although some predetermined specific and difficult goals can improve performance in most of patients [39].

Another question is represented by the super specialization of the existing robots, most of which focus on the proximal (MIME, T-Wrex, and Arm-Guide) or the distal (Master II) part of the upper limb: just ADLER, allows the training of the whole arm (shoulder, elbow, forearm, wrist, and hand). Moreover, at the present time robot training towards the full range of joint motion and the possible degrees of freedom is not possible. This aspect might explain the lack of functional recovery in the daily life, which is based on the use of the whole arm, not just the shoulder or the elbow. As Reinkensmeyer and Boninger [40] recently underlined, two opposite tendencies are accredited worldwide, with some groups working on simpler technology, while others developing more sophisticated mechanical devices. The aim of a simpler technology is to promote the widespread diffusion of more economic and easier robotic devices, also in a home setting. On the other hand, the creation of more sophisticated devices allowing a full range of motion should improve functional outcomes. Future devices will probably include the main features of the two different options.

An important potential advantage is that robotic systems can measure several kinematic and dynamic parameters during the motion of the patient's limb, allowing for both online and offline evaluation of relevant indicators of the patient performance (e.g., range of motion, speed, smoothness, etc.) [41]. These values can be used to rate the patient's progress more objectively than the clinical evaluation scales. On the other hand, the engineering parameters proposed so far, usually related to the specific robotic hardware employed and/or to the type of exercise implemented, are not suitable as alternative indicators to the traditional evaluation scales.

The acceptance of robotic technology by patients and physiotherapists may be an issue itself, although this does not represent a major concern for the devices developed to date. Moreover, the cultural gap between technology providers, rehabilitation professionals and final users is becoming gradually smaller thanks to the widespread diffusion of knowledge in the recent past.

Regarding the lower limb, there is evidence of beneficial effects of electromechanical devices for gait training after stroke, but their relatively high cost limited the diffusion of such devices in the clinical practice. Hence, more evidence about the cost effectiveness of such technology is required, in order to warrant their broad clinical application.

In the international context there is an open discussion on which kind of electromechanical gait training device is more effective. A recent review study [42] including 18 trials, investigating differences between types of electromechanical training devices (end-effectors and exoskeleton devices), found that an end-effector approach may be more favourable for gait training after stroke, although the reason for this superiority are not clear yet. According to the authors, future research should include randomized clinical trials, comparing the effectiveness of exoskeleton and end-effector electromechanical devices.

## 4. Conclusions

After pointing out the areas of agreement and those of controversy in the field of robotic rehabilitation after stroke, we conclude illustrating the currently growing points and the areas timely for development research.

### 4.1. Growing Points

Robotic rehabilitation is certainly undergoing a period of rapid growth, although many issues remain open. However, this technique may offer considerable benefits, not only in terms of cost reduction and training program implementation, but also for the scientific community with an approach inspired from evidence-based medicine. Robotic technology, indeed, differently from other physiotherapy options, allows quantifying objectively the amount and quality of multisensory stimuli and measure patient outputs and outcomes (e.g., times, movements, coordination, and strength improvement).

Present researches in this field are trying to improve the efficacy of robotic manipulation, the trajectories, strengths, and multisensorial inputs that robots must provide in order to improve the quality and efficiency of rehabilitation. In the last years, very different robotic systems and approaches were employed for rehabilitation treatment of impaired upper and lower limbs in poststroke patients. The future systems should comply with principles of motor learning mentioned below [11, 26].

1. The modality in which the subject performs: brain stimuli and motor gain seem to be greater with intense, active, assistive, and repetitive movements than with nonassistive or passive movements.
2. The graduation of amount and typology of feedback (visual, auditory [43], haptic feedback) in relation to the degree of active subject movements, or to the degree of patient's attention or active participation: there is still a lack of knowledge on the actual relation between sensory information and patient engagement and effort. This relation should be further investigated to implement the novel robotic systems for rehabilitation, also integrating concepts of the virtual reality.
3. The multiplanarity of the exercises that seems to induce more motor cortex excitation.

In poststroke patients with upper arm impairment robotic devices can be applied in the acute, subacute, and chronic phases. Robot therapy was introduced only in the chronic phase of the treatment protocol in most of the studies published so far. However, the application of this approach in the acute or subacute phase of stroke may lead to a clinical improvement, mainly due to the increased brain plasticity earlier after stroke [21, 44]. Even if the early mobilization after stroke has been widely accepted as fundamental, there is still a lack of rehabilitation interventions in stroke units [44].

At the present time many scientific efforts are addressed towards the construction of handy and easily transportable devices suitable for a robot assisted training in the acute and subacute phases, such as the wearable technology [26, 45]. In a recent review Reinkensmeyer and Boninger [40] state that the rationale for developing actuated, wearable orthotic systems is to make training more naturalistic and ultimately to release training from the confines of the rehabilitation field, breaking down the current distinction between assistive and therapeutic technology; thus therapeutic technology can be applied to assist people in ADLs, discontinuing therapy after the achievement of the desired tasks.

The research in wearable technology is oriented not only to stroke patients, but also to other patients with limited mobility [46]. Hopefully, in the near future robotic devices will be available to clinicians, both in the hospital setting and in a home-based context. Another long-standing issue is represented by robotic hand rehabilitation, in which several working groups are committed, with still preliminary results [47–49].

### 4.2. Areas Timely for Developing Research

Given the fast developments in rehabilitation technology, it is mandatory to understand the role of robotics in the rehabilitation program. Our review confirms the theory reported in the commentary of Johnson et al. [50] that technology for supporting upper limb training after stroke should take into account the recent interest in rehabilitation towards functionally oriented targets. According to the present literature, it is not yet understood how different rehabilitation approaches may contribute to restorative processes of the central nervous system after stroke. Although some rehabilitation technology approaches show promising results in small studies, it would be interesting to test these hypotheses on randomized clinical trials.

Robot-mediated neurorehabilitation is a rapidly advancing field based on robotics, virtual reality, and haptic interfaces that could support neuroscience and conventional rehabilitation for a successful treatment of neurological injuries such as stroke, spinal cord injury, and traumatic brain injury. According to the latest research a composite approach including robotics, virtual reality [51, 52], and transcranial direct current stimulation (TDCS) [53, 54] should be adopted in neurorehabilitation.

Machine-mediated neurorehabilitation is characterized by challenges both in engineering and in clinical practice. From a technical point of view, there is a need for more integrated solutions to perform a therapy in a safe environment and with better compliance from the patients. According to the current practice, new machines patented for rehabilitation use must be tested clinically, and the results published. More research is needed to establish whether ADL tasks can be truly enhanced by robotic training. Moreover, solutions improving the interaction between the therapist and patients in the robotic field are mandatory. As a recent review recommends, methodology in trials including robotic resources should be unified, especially in terms of inclusion criteria according to the poststroke phase, functional scales for outcome measure, intensity, and duration of the interventions [55]. Ashford et al. [56] could not establish a single valid and reliable outcome measure in order to rate the function of paretic upper limbs. During the acute phase of stroke the robotic device must be available bed-side in hospital, to a possibly unresponsive patient, while in chronic phase, when the patient visits the rehabilitation gymnasium, either as an inpatient or outpatient setting, it has to be more specifically orientated to limbs' movements. Patients usually take advantage from an early discharge and from the prosecution of rehabilitation in a home setting that is also a cost-effective measure. However, self-training of the patient with the robotic equipment in a home setting should be avoided. Therefore, discharge from hospital should not be justified when home rehabilitation is performed self-directed and with little professional feedback to resolve this issue; the outpatient setting could be proposed and new modalities based on telerehabilitation might be an appropriate resource for the home setting.

Moreover, the successful clinical implementation of this promising field meets opposition from the concerns raised about the possibility that robots could “dehumanize” patient rehabilitation or replace the human work force. In conclusion, these machines are intended to represent an adjunctive tool to increase the intensity of therapies, in line with modern principles of motor rehabilitation. A robot can never replace both the multilevel interactions between patient and experienced physical therapist and the manual ability of operators.

## Figures and Tables

**Table 1:** Upper limb robotic systems classification.

Classification	Characteristics
According to the part of the upper limb on which the therapy is focused, there are robots specifically designed for:	(1) Unilateral or bilateral shoulder movement (2) Elbow movement (3) Wrist movement (4) Hand movement

According to their mechanical characteristics, rehabilitation robots can be classified into at least two main groups:	(1) Exoskeleton (2) Operational machines/*end-effectors *

According to the control strategy, robots can be programmed to deliver different exercises. In fact, robotic systems are capable of assisting the motion of the patient in a number of different modes:	(1) Passive movement in which the robotic device moves the patient's arm. (2) Active nonassist mode in which the subject executes the exercise and the robot provides no help. (3) Active assist mode in which the subject attempts to move, and the robot provides assistance when there are some voluntary but inadequate movements. (4) Resistive mode when the subject is required to perform an exercise against an antagonist force provided by the robot. (5) Bimanual exercise in which active movement of the unaffected arm is mirrored by simultaneous active/passive/assistive movement of the affected arm by means of the robotic device.

**Table 2:** Main electromechanical and robot-assisted arm training devices.

Devices	Characteristics
InMotion robot	3 active degrees of freedom (DOFs) wrist robot mounted at the tip of a companion planar robot (MIT-MANUS), allowing 5 active DOFs at the shoulder, the elbow, and the wrist.

Mirror Image Movement Enhancer	6 DOFs robot manipulator; the treatment focused on shoulder and elbow function; unilateral or bilateral upper limb training.

Bi-Manu-Track	1 DOF system to train forearm pronation/supination and wrist flexion/extension; bilateral training in passive or active mode; no feedback to the patient.

Gentle/S	3 DOFs robot manipulator (HapticMaster, FCS Robotics, The Netherlands) with an extra 3DOF passive gimbal mechanism (allows for pronation/supination of the elbow as well as flexion and extension of the wrist), an exercise table, computer screen, overhead frame and chair.

Arm robot ARMin	Semiexoskeleton for movement of the shoulder (3DOFs), the elbow (1DOF), the forearm (1DOF), and the wrist (1DOF); matched with an audio-visual display used to illustrate the movement task to the patient.

Assisted Rehabilitation and Measurement Guide	4 DOFs robotic device provides arm reaching therapy for patients with chronic hemiparesis; it gives patient a real time visual feedback of the location of the arm.

REHAROB Therapeutic System	Firstly for rehabilitation robotics, uses standard industrial robots, not modified, but equipped with extra safety systems and a special instrumented orthotic, developed for fixing the patient's limb it provides passive shoulder and elbow physiotherapy. limb; it provides passive shoulder and elbow physiotherapy.

NeuroRehabilitation Robot	3 DOFs robot, based on direct-drive wire actuation; it gives patient visual and auditory feedbacks; easily transportable.

**Table 3:** Main automated electromechanical gait machines.

Classification	Devices	Characteristics
Robot-driven exoskeleton orthotic	Lokomat	Robotic gait orthotic combined with a harness-supported body weight system used in combination with a treadmill. The robotic device according to a preprogrammed gait pattern guides patient's legs; the process of gait training is automated.
	LOPES Lower- extremity powered exoskeleton	Combination of a freely translatable and 2-D-actuated pelvis segment with a leg exoskeleton containing three actuated rotational joints: two at the hip and one at the knee.

End-effectors (electromechanical solutions with two driven foot plates simulating the phases of gait)	Gait Trainer GT-1	Two foot-plates symmetrically simulate the stance and the swing phases of walking while the patient is on the devices.
	Haptic walker	Evolution of the “Gait trainer GT-1”: it allows simulation of walking up-or downstairs; the walking speeds can be fully adjusted to individual patients' needs; it has 6 DOFs force/torque sensors located under each footplate; it can be integrated into virtual GT environments and combined with other modalities (e.g., visual feedback).

	Lokohelp	It is a device that can be placed on a treadmill, easily installed and removed: it transmits the treadmill movement to levers positioned on both sides of the device, so the simulation of gait is achieved by the track of the levers, which imitate the stance and swing phases.
	G-EO-System	It consists of two foot-plates, freely programmable; its main characteristic is to enable not only the practice of simulated floor walking, but also stair climbing up and down.
